# Autoimmune hepatitis type 2 associated with an unexpected and transient presence of primary biliary cirrhosis-specific antimitochondrial antibodies: a case study and review of the literature

**DOI:** 10.1186/1471-230X-12-92

**Published:** 2012-07-20

**Authors:** Pietro Invernizzi, Maria Grazia Alessio, Daniel S Smyk, Ana Lleo, Aurelio Sonzogni, Luca Fabris, Manila Candusso, Dimitrios P Bogdanos, Raffaele Iorio, Giuliano Torre

**Affiliations:** 1 Center for Autoimmune Liver Diseases, Humanitas Clinical and Research Center, Via Manzoni 56, 20089, Rozzano(MI), Italy; 2 Division of Rheumatology, Allergy and Clinical Immunology, University of California at Davis, Davis, CA, USA; 3 Clinical Laboratory, Ospedali Riuniti, Bergamo, Italy; 4 Institute of Liver Studies, King’s College London School of Medicine at King’s College Hospital, Denmark Hill Campus, London, UK; 5 Department of Medicine and Transplantation, Ospedali Riuniti, Bergamo, Italy; 6 Department of Surgical and Gastroenterological Sciences, University of Padova, Padova, Italy; 7 Center for Liver Research (CeLiveR), Ospedali Riuniti, Bergamo, Italy; 8 Division of Pediatrics, Ospedali Riuniti, Bergamo, Italy; 9 Department of Pediatrics, Federico II University, Naples, Italy

**Keywords:** Autoantibody, Autoimmunity, Autoimmune cholangitis, Epidemiology, Environment, Paediatric liver diseases.

## Abstract

**Background:**

Unlike other autoimmune liver diseases, primary biliary cirrhosis (PBC) has never been reported in early childhood, while type 2 autoimmune hepatitis (AIH) is eminently a paediatric disease.

**Case presentation:**

We describe a case of type 2 AIH with serological positivity for PBC-specific anti-mitochondrial antibodies (AMA) in a 3-year old girl. We found this observation intriguing as AMA and indeed an overlap with PBC are virtually absent in Type 2 AIH, a pediatric form of AIH which is distinct precisely because it is characterized by pathognomonic anti-liver kidney microsomal type 1 (LKM-1) showing a remarkable antigen-specificity directed against cytochrome P4502D6. We also review the literature in relation to AMA positivity in paediatric age and adolescence. In our case, the presence of AIH-2-specific anti-LKM-1 and PBC-specific AMA was confirmed by indirect immunofluorescence (IIF), and immunoblotting and ELISA based on recombinant mitochondrial antigens. The clinical, laboratory and histological features of the child are given in detail. Interestingly the mother was AMA positive without other features of PBC. The child was successfully treated with immunosuppression and five years after the original diagnosis is on a low dose of prednisolone and azathioprine, with no signs of relapse. Anti-LKM-1 antibodies are still present in low titres. AMA were detectable for the first 4 years after the diagnosis and disappeared later.

**Conclusion:**

This is the first case report in the literature of AIH type 2 with an unexpected PBC-specific AMA positivity in a young child. Response to immunosuppressive treatment was satisfactory and similar to that described in AIH. A review of published reports on AMA positivity in paediatric age shows that the antibody may arise in the context of immunodeficiency and is variably associated with liver damage.

## Background

Primary biliary cirrhosis (PBC) typically affects middle aged women and has never been reported in previously healthy young children, although affected adolescents have been described [1-22]. The disease is characterized by an immune-mediated destruction of intrahepatic bile ducts and the presence of high-titer anti-mitochondrial antibodies (AMA) against the E2 subunit of the pyruvate dehydrogenase complex (PDC-E2) [3,9,11-13,23-39]. AMA are highly specific for PBC, and can be detected in approximately 95% of patients when sensitive diagnostic immunoassays are used [3,6,9,11-13,18,21,23-38,40-50]. The etiology of the disease remains elusive, but is believed to derive from a combination of factors including a multi-lineage loss of immunological tolerance to PDC-E2 [10,13,15,16,29-31,33,36,43-45,51-84], genetic susceptibility [6,9,11,22,51,77,85-111] and exposure to environmental triggers [51,52,58,112-135]. Descriptive epidemiological studies strongly suggest that the incidence and prevalence of PBC are increasing [136-140].

We herein describe the unusual case of a 3-year-old girl with overlapping autoimmune hepatitis type 2 (AIH-2) and PBC-specific AMA positivity. She presented with acute liver failure with no evidence of infections, metabolic and genetic liver disease or other causes of acute liver disease. The autoantibody testing revealed the presence of anti-liver kidney microsomal antibodies (LKM-1) and PBC-specific AMA, the autoantibody markers of AIH-2 and PBC, respectively. Though histological findings did not demonstrate typical overlap of PBC and AIH, they were compatible with both AIH and overlapping biliary features. AMA were also detected in the mother’s serum samples.

Over a seven-year period, 3808 paediatric patients with liver disease were screened in a single tertiary center in Northern Italy, Bergamo, for the presence of serum autoantibodies. The screening program included children with acute or chronic liver diseases in the course of evaluation for inclusion onto a waiting list for orthotopic liver transplant (OLT), or during follow-up after OLT. Throughout that period, 340 paediatric OLT were performed. Out of 3808 tested patients, only 2 tested positive for AMA by indirect immunofluorescence (IIF), which was confirmed by western blot with recombinant antigens. The first one presented with acute liver failure (ALF) with massive hepatic necrosis, was transplanted and spontaneously lost AMA after OLT. The second one is herein described. To our knowledge, this is the first child with AIH type 2 and AMA positivity documented at the level of individual PBC-specific mitochondrial antigens ever reported.

## Case report

SM, a 3-year-old previously healthy girl, came to medical attention because of progressive jaundice, fatigue and anorexia. She was admitted to a local hospital and on examination was found to be icteric with hepato-splenomegaly but no ascites. No prior history of early deaths, liver disease or autoimmunity existed in the family. Laboratory tests indicated cholestatic hepatitis without impaired liver function (Table1). However, her condition deteriorated within 24 hours with features of acute liver failure (International normalized prothrombin ratio [INR] 2, increasing hyper-ammoniemia from 55 to 105 and up to 196 mMol/L) and neurological deterioration. Five days later, she was admitted to the Pediatric Liver Transplant Centre (Ospedali Riuniti, Bergamo) with stage I hepatic encephalopathy. Ultrasound examination demonstrated a hyper-echogenic left hepatic segment with structural alteration, suggestive of chronic parenchymal damage.

**Table 1:** Routine laboratory results before, at admission and during previous outside hospitalization. Therapy with steroids and cyclosporine was started on day 1

	**t = −5 days**	**Admission t = 0**	**t = 20 days**	**t = 40 days**
AST (IU/L) nv < 35	3,700	1,243	128	25
ALT (IU/L) nv < 32	2,800	1,249	234	47
Bilirubin, total (mg/dl) nv < 1.2		16.90	7.90	1
Bilirubin, direct (mg/dl) nv < 0.3	10	14.60	5.50	0.60
Albumin (g/dl) nv < 3,5-5 g/dl		4.20	3.66	4
INR nv 0.9-1.2	2.00	2.15	1.23	1.07
Immunoglobulins				
IgG (mg/dl) nv 707–1919	nt	2230	1020	nt
IgA (mg/dl) nv 60–270	nt	271	136	nt
IIgM (mg/dl) nv 61-276	nt	200	122	nt

t, time point; nv, normal value; nt, not tested.

AMA and LKM were strongly positive (> 1:640), and total serum IgG and IgM levels were elevated. The laboratory tests excluded viral infections (hepatitis virus A, B, C, D, human immunodeficiency virus, Epstein-Barr, cytomegalovirus, and herpes simplex virus), metabolic and genetic alterations (Wilson disease, haemochromatosis, and alpha-1 antitrypsin deficiency). Liver biopsy was contraindicated because of the coagulation abnormalities.

Based on the clinical and laboratory findings, i.v. therapy with methylprednisolone (2 mg/kg/day) and cyclosporine (continuous infusion at doses of 2–3 mg/kg/day in order to maintain a blood level up to 300 ng/ml) was started. During the following 36 hours, hepatic encephalopathy progressed to stage III and hepatic function deteriorated (Table1), and the child was listed for urgent OLT. However the child improved in the following days with complete neurological recovery and she was removed from the transplant list. One week after the beginning of therapy, the hepatic function was normal, and steroid tapering was initiated one month later. In the following 3 months, steroids were decreased and cyclosporine was switched to azathioprine (50 mg/day).

**Figure 1:**
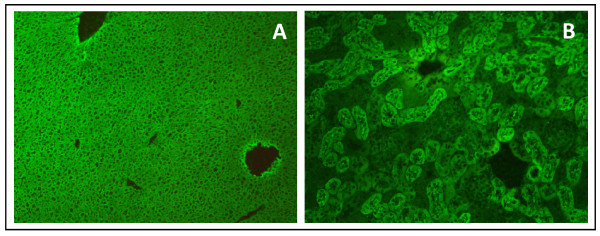
Autoantibody testing by conventional indirect immunofluorescence of the serum of the child on rat liver and kidney sections showing a typical staining of the liver cytoplasm (A) and renal tubules (B) corresponding to that seen by liver kidney microsomal type 1 autoantibodies.

**Figure 2:**
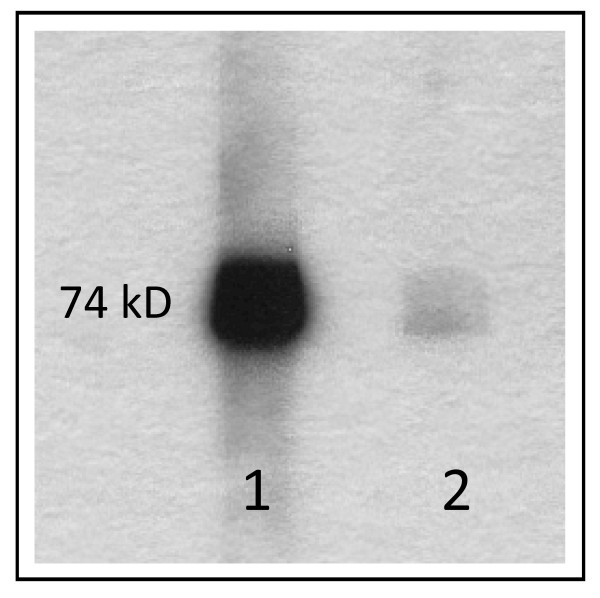
The presence of AMA was determined by Western-blot with recombinant mitochondrial PDC-E2. Serum from the patient (1) and the patient’s mother (2) were tested at 1:500 dilution.

Repeated laboratory tests confirmed the presence of both anti-LKM-1 antibodies by IIF (Figure1) [48,141,142] and AMA by western blot with recombinant antigen [13] (Figure2) over a two year period. Seropositivity for PBC-specific autoantibody responses were also confirmed using an a PBC profile ELISA based on a mixture of the triple MIT3 hybroid and PBC-specific ANA gp210 and sp100 peptides (Quanta Lite PBC profile, INOVA Diagnostics, San Diego, California, USA). Serum samples were negative for PBC-specific ANA by IIF. At the time of the diagnosis, both parents were alive and in good health, and no autoimmune diseases were reported in first grade relatives. Sera from the parents, maternal and paternal grandparents and one maternal uncle were also collected and tested for the presence of autoantibodies. The child’s mother was found to be positive for AMA directed against the major PBC-specific mitochondrial autoantigen, but had no symptoms or signs of liver disease (Figure2).

A percutaneous liver biopsy was performed 4 weeks after presentation, when the INR normalized. Ductular structures were stained using cytokeratin 7 (CK-7) (NeoMarkers, Freemont, USA, working dilution 1:200), a marker selectively expressed by the biliary epithelial cells in the human liver. Histological examination showed a normal liver architecture with mild mixed inflammatory infiltrate and minimal portal fibrosis. Immunohistochemical staining for CK-7 demonstrated marked proliferation of medium and small sized bile ducts in portal and periportal areas. Focal hepatocytes demonstrated biliary metaplasia which was evident by the same immunohistochemical staining. Mixed steatosis was observed in about 20% of liver cells (Figure ss3). Portal tracts were expanded, fibrotic and oedematous, with a moderate inflammatory infiltrate, mainly represented by lymphocytes with spill-over features and isolated granulocytes. Interlobular bile ducts were affected by destructive lesions with focal destruction of basal membrane by inflammatory cells, occasionally migrating within the biliary epithelium. Typical histological lesions of PBC are difficult to be seen in the context of concomitant features of acute severe hepatitis related to AIH. It is possible that the first cycle of corticosteroid therapy had a stronger effect in ameliorating the hepatitis component, thereby allowing the cholangiopatic component (which is less likely to be responsive to corticosteroid therapy) to become more evident once the subacute lesions disappeared.

**Figure 3:**
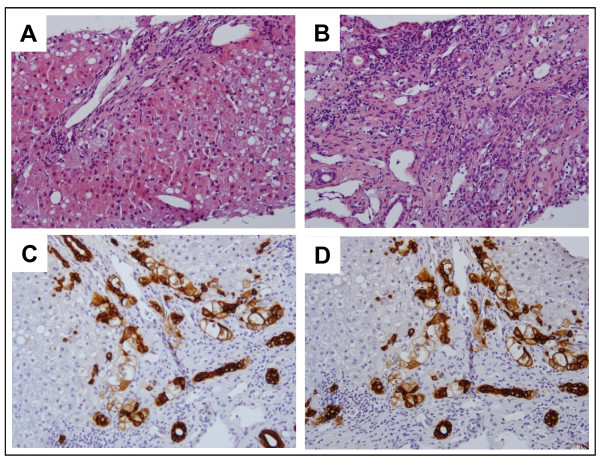
**Representative analysis of liver tissue sections. Portal and periportal cellular inflammation: lymphocytes, monocytes/macrophages and plasma cells infiltrate the portal tracts and invade the surrounding parenchyma, resulting in the characteristic picture of interface hepatitis.** (**A**) Enlarged portal tracts fields with fibrosis and marked bile ductular proliferation, severe inflammatory biliary tract damage; liver cells showing moderate microvescicular steatosis (H&E, 20X); (**B**) high-power view of an expanded portal tract field with moderate mixed inflammatory infiltrate and some features of flogistic bile duct damage (H&E, 40X). (**C** and **D**) Immunohistology for biliary type cytokeratin 7 shows marked ductular proliferation in portal tracts fields and biliary metaplasia (abnormal positivity to cytokeratin 7) of peri-portal hepatocytes (40 X).

Five years after the acute episode, the child is on a low dose of steroid (prednisone 5 mg/day) and azathioprine (50 mg/day), with normal liver function. AMA and anti-LKM-1 remained positive for four years; in the course of the 5th follow up year, AMA became undetectable while anti-LKM-1 remained positive (January 2011).

## Discussion

We herein present the rare finding of type 2 AIH with the unexpected presence of AMA. Unlike other autoimmune liver diseases, typical features of PBC have never been reported in early childhood. Reported paediatric autoimmune liver disorders include AIH types 1 and 2, and autoimmune sclerosing cholangitis (ASC) [17]. The diagnosis of ASC relies on cholangiographic imaging changes. AIH type 2 is less frequent than AIH-1, affects mainly children and young adults and has a more aggressive course, leading to fulminant hepatic failure more often than type 1 AIH. In general, AIH responds satisfactorily to immunosuppressive treatment, with AIH-2 usually requiring treatment for life. The positive response to immunosuppression of the present case, in addition to the high titer of anti-LKM-1 antibodies, strongly supports the AIH component of the disease. Anti-LKM-1 antibodies in AIH-2 are specifically directed against cytochrome P4502D6. In addition to AIH-2, anti-LKM-1 is present in a minor proportion of chronic HCV-infected patients. HCV infection was ruled out in the present case of AIH-2. A diagnosis compatible with PBC has been considered on the basis of the presence of PBC-specific AMA, evidence of cholestasis and histological lesions - not typical of PBC such as the granulomatous destruction of septal or interlobular bile ducts, but indicative of portal tract inflammation and ductular damage [13].

The presence of PBC-specific AMA in this case is intriguing. AMA are highly specific for PBC, and often precede the development of liver damage by several years, even in individuals who are asymptomatic and do not have any other evidence of chronic liver disease [13]. Although the mechanisms leading to the generation of AMA are unknown, it has been postulated that xenobiotic-induced and/or oxidative modification of mitochondrial autoantigens is a critical step leading to loss of immunological tolerance.

### Paediatric PBC

Another intriguing finding of the present case is that PBC-specific AMA was present in conjunction with evidence of biliary epithelial cell destruction, diagnosed at the age of 3. The presence of AMA in the present case raises the question as to whether PBC has been reported among paediatric patients. The youngest child previously described with PBC was a six year old female [35] (Table2). The remaining cases of ‘paediatric’ PBC have been described in adolescents [1,2]. Dahlan and colleagues reported two females aged 11 and 16 years, with confirmed PBC [2]. The first patient presented at 11 years of age with abdominal pain and raised aspartate aminotransferase (AST) (48 U/L; normal values <35 U/L), but all other liver biochemistry tests (including ALP) were normal (γGT was not tested) [2]. Her abdominal pain continued and liver function tests demonstrated a raised γGT (107 U/L) at the age of 15, when she was found to be positive for AMA by IIF (1:800), although the specificity of the AMA was not stated [2]. No other autoantibodies were detected [2]. Her serum IgG and IgA were normal but IgM was raised [2]. A liver biopsy at age 16 showed stage II PBC (damaged segmental bile ducts with portal and periportal lymphoid infiltrates), and liver function tests at age 18 demonstrated a cholestatic profile with raised ALP (660 U/L) and slight increase of total bilirubin (24 μmol/L) [2]. It was at this phase that she developed symptoms of pruritus, fatigue and weight loss [2]. She was transplanted at age 21 following worsening of symptoms and increasingly abnormal liver biochemistry despite ursodeoxycholic acid (UDCA) treatment [2]. Histology of the explanted liver demonstrated stage IV PBC. An intriguing aspect of that case is her strong family history of liver disease, as the mother presented at the age of 30 with overlapping features of PBC and AIH, and received a liver transplantation at the age of 34 [2]. Additionally, the grandmother and great-grandmother on the maternal side died of liver cirrhosis of unknown origin [2]. This is of interest given that the mother of the child in the current report is also AMA positive, suggesting a possible genetic predisposition. In the second case, a 16 year old female presented with Raynaud’s and Sicca syndromes as well as raised AST (163 U/L), and was found to be positive for AMA (1:160). She was also positive for ANA (1:320) but it is not clear whether these ANA were those specific for PBC. (i.e. with a multiple nuclear dot or a rim like membranous pattern) [2]. Serum IgG and IgA were normal, but IgM was raised [2]. Histology confirmed stage II PBC at the age of 17 years, and she was started on UDCA (2 g/day), with improvement of her symptoms and liver biochemical tests [2].

**Table 2:** Characteristics of paediatric patients with antimitochondrial antibody (AMA) positivity in primary biliary cirrhosis (PBC) and other liver or non-liver related diseases

**Reference**	**Age**	**Sex**	**Pathology**	**Antibodies Detected**	**Histology**
Zamfir et al.	6 yrs	M	ITP	AMA, ANA	-
	15 yrs	M	AHA	AMA, ANA	-
Gregorio et al.	12 yrs	F	AIH	AMA	Portoseptal mononuclear cell infiltration and extensive interface hepatitis, with lymphocytic periportal necrosis. Consistent with chronic hepatitis.
Hannam et al.	Birth	F	NNH	AMA	
	Birth	M	NNH	AMA	Cholestasis, hepatitis, mild cholangiolitic changes and multi-nucleated giant hepatocytes. Mild portal fibrosis.
Aoki et al.	6 months	M	IL-2Rα deficiency	AMA	Intense mononuclear lymphocyte infiltration of the portal tracts with preservation of the lobular architecture.
Tsuda et al.	11 yrs	M	IPEX	AMA	-
Melegh et al.	6 yrs	F	PBC	AMA	*At 6 years*: Fibrotic degeneration of the portal tracts, loss of bile ducts, ductal proliferation, periportal hepatocytes separated by mononuclear inflammatory cells.
Dahlan et al.	11 yrs	F	PBC	AMA	*At 16 years*: Stage II PBC *At 21 years*: Stage IV PBC (explanted liver)
	15 yrs	F	PBC	AMA, ANA	*At 17 years*: Stage II PBC

Detailed description of cases is given within the text. ITP: Idiopathic thrombocytopaenic purpura; AHA: Autoimmune haemolytic anaemia; AIH: Autoimmune hepatitis; NNH: Neonatal hepatitis; IPEX: Immunodysregulation polyendocrinopathy enteropathy X-linked syndrome; ANA: Antinuclear antibody.

### Paediatric ‘PBC’ due to genetic deficiences: The case of IPEX and IL-2

Immunological and histological features of PBC have been reported in younger children, but these extreme cases occur due to underlying genetic deficiencies such as IPEX syndrome or IL-2 receptor alpha (IL-2Rα) deficiency [37,143]. IPEX syndrome is a congenital disorder of immune regulation caused by mutations in the FOXP3 gene, which is required for the suppressive function of naturally arising CD4 + CD25 + regulatory T cells [143-145]. Tsuda et al. [37] note that patients with IPEX syndrome produce a variety of autoantibodies including AMA. Mutations in the FOXP3 gene (located on the centromeric region of the X chromosome) lead to decreased CD4 + CD25+ Tregs [63,64,146], and therefore failure to suppress the production of autoreactive T cells and multi-organ autoimmunity [37]. Tsuda and colleagues note that one individual in their study, an 11 year old male with IPEX, was positive for AMA, with no clinical or biochemical evidence of liver disease [37]. The AMA in that case was of the IgA isotype, with no AMA of the IgG isotype identified, and the plasma IgM and IgA levels were also raised [37]. It was not stated whether the AMA in that case was directed against PDC-E2, and it is unknown whether the child in that case eventually went on to develop PBC [37]. Aoki et al. [143] report the case of a male child born to consanguineous parents, who initially presented with recurrent infections at six months of age. At five years of age, liver biochemistry showed an elevated GGT, and a liver biopsy demonstrated mononuclear lymphocyte infiltration of the portal tracts [143]. These findings differ from the typical histological appearance of PBC, which includes non-suppurative cholangitis, ductopaenia, and sporadically non-caseating granuloma formation. The infant also had CD3+ CD25+ lymphocytopaenia leading to an abnormal CD4:CD8 ratio [143]. Infectious causes were ruled out, but antibody testing was positive for AMA, with reactivity to PDC-E2. Immunoblotting of peripheral lymphocytes showed the absence of the IL-2Rα. A thymic biopsy showed absent CD1a and increased Bcl-2 (an anti-apoptotic protein) expression, due to a four point mutation in FOXP3 leading to translational frameshift, which is similar to IPEX syndrome [143]. The authors suggested that the lack of CD4 + CD25+ Tregs led to a proliferation of autoreactive T cells which were not induced to apoptose due to an increased Bcl-2 [143]. In turn, these autoreactive T cells contributed to the development of PBC (or PBC-like pathology), likely via close interaction with B cells [143,144]. The child in this case underwent myeloablative chemotherapy and allogenic stem cell transplantation, with complete resolution of his symptoms and no residual serological reactivity to recombinant PDC-E2 [143]. As mentioned, these cases do not represent true paediatric PBC, as the underlying pathological process was linked to a genetic deficiency.

### Antimitochondrial antibodies in liver disease-free paediatric cases

The presence of AMA is highly suggestive of PBC, or predicts progression of an asymptomatic patient to overt PBC [13,36]. However, Zamfir et al. [83] noted the presence of AMA in 10 of 900 patients presenting with extrahepatic disorders. Of those patients, nine were presenting with haematological disease and one with a dermatological condition [83]. Eight of the ten had high AMA titres, with the remaining two having low titres, but the presence of AMA was confirmed by immunoblot. The immunodominant antigen in all positive AMA cases confirmed by immunoblot was PDC-E2 [83]. It is not known if there was also reactivity to other PBC specific AMA antigens such as the E2 subunits of branched chain oxoacid dehydrogenase complex (BCOADC-E2) and oxoglutarate complex (OGDC-E2) [13,26]. Interestingly three of the 10 patients were under the age of 20, at 19, 15 and 6 years of age [83]. All three of these individuals were male, and had significant AMA titres, as well as positive ANA of unknown specificity [83]. The presenting disorders among the three were haematological, autoimmune haemolytic anaemia, thrombocytopaenia and idiopathic thrombocytopaenic purpura, respectively [83]. It should be noted that in the case of the 15 year old, the AMA appearance by IIF were atypical (non-anti-M2), and the positivity was not confirmed by immunoblot. It is therefore reasonable to speculate that this case may represent a non-PBC related AMA or a false positive AMA test. The 19 and 6 year old cases had AMA titers of greater than 1/640, and the presence of AMA was confirmed by immunoblotting [83]. These patients were unusual not only due to their young age, but also because they were male. Of the remaining 7 AMA positive patients with extrahepatic disorders, six were female [83]. It has been suggested that these patients may eventually go on to develop PBC, but whether this occurred or not is unknown, as no liver biopsy or follow-up studies were performed.

### Antimitochondrial antibodies in cases with non-PBC related liver disease

Unlike the above study where no liver disease was reported in the presence of high AMA titres, Gregorio et al. [26] report a 12 year old girl with autoimmune hepatitis type 1 who was AMA anti-M2 positive. This patient initially presented with a four month history of jaundice, fatigue, anorexia and weight loss, and on examination was found to have hepatosplenomegaly and liver disease stigmata [26]. Her serum bilirubin and ALT were elevated, but all other liver biochemistry was normal. She had increased IgG and was positive for PBC-specific anti-M2 AMA (titre 1/640) [26]. The presence of anti-M2 AMA was confirmed by western blot analysis [26]. Immunological testing for ANA, anti-smooth muscle antibody, and anti-LKM-1 were negative [26]. Histological assessment of six liver biopsies taken over several years showed chronic hepatitis with varying degrees of inflammation, but no evidence of PBC [26]. Treatment with prednisolone and azathioprine improved her condition, and her transaminases normalised, but she had three episodes of spontaneous bacterial peritonitis and four episodes of relapse due to poor treatment compliance [26]. Twelve years after her initial diagnosis, she developed liver failure and died following an intraperitoneal haemorrhage. Liver tissue obtained at autopsy confirmed cirrhosis with persistent inflammation, but no features confirmatory of or compatible with PBC [26]. Over the twelve years since diagnosis, she remained AMA positive, with titers ranging from 1/40 to 1/1280. She did not develop Sicca syndrome, systemic sclerosis or any other rheumatological condition associated with AMA positivity or PBC [26]. Her low IgM and ALP, as well as the lack of histological PBC features, are atypical for “overlap” between autoimmune hepatitis and PBC seen in adults [13,26,142].

### Transplacental passage of AMA and neonatal liver disease

Hannam et al. [28] report two cases of neonatal liver disease, in which the transplacental passage of AMA occurred. The first case was that of a female infant born to a 32 year old mother with a three year history of pruritus and joint pain, who was AMA positive with normal liver function tests. The foetus was found to be hydropic, and required three intrauterine transfusions before delivery at 31 weeks by caesarean section [28]. The infant displayed deranged liver function tests, and was positive for AMA (titre 1/160) and ANA (1/20) [28]. These autoantibodies were present in the mother at the same titre, and both had the same antibody epitope recognition pattern to PDC-E2 and PDC-E3 binding protein (also known as PDC-X), recognising the major epitopic regions on these antigens [28]. Over several weeks the infant’s condition and liver function tests improved, and the autoantibodies became undetectable. The second case involved a male infant born to a 28 year old mother following an uneventful pregnancy. The infant presented at five weeks with poor weight gain, jaundice, pale stools and an erythematous rash [28]. Liver function tests were deranged, and an antibody screen was positive for AMA (titre 1/20) and ANA (1/10) [28]. The mother was also positive for the same antibodies at the same titre, but she was asymptomatic and had normal liver biochemistry [28]. Titres of 1/10 and 1/20 based on IIF using liver, kidney, stomach tissues are considered significant in children, though in adults titers are considered positive if they exceed 1/40. Antibodies in both mother and child had an identical epitope recognition pattern to PDC-E2 and PDC-E3 binding protein [28]. A liver biopsy of the infant demonstrated cholestasis with hepatitis and mild portal fibrosis [28]. Over the course of three months the infant's condition improved, liver biochemistry normalised, and the antibodies became undetectable [28]. In both cases the AMA were demonstrated to be maternal in origin, as in both infants they were of the placenta-crossing IgG1 and IgG 3 class, their concentrations declining over several weeks [28]. The epitope recognition pattern was also the same in both infant-mother pairs [28]. These cases are of interest given the transplacental passage of AMA, and its association with liver disease in the infants. Additionally, the AMA positivity was transient, akin to the current case report.

### Transient AMA positivity in acute liver failure

In the patient herein illustrated, AMA became undetectable five years after the initial presentation. The decline of AMA over time may be due to the effect of immunosuppressive treatment or may indeed reveal the transient nature of the AMA positivity. A transient appearance of AMA has been previously noted in North American patients with acute liver failure (ALF). AMA have been reported to be present in up to 40% of adult patients with ALF and no other signs of PBC [32]. One study examined 217 serum samples from 69 patients with ALF, with samples collected over a 24-month period [32]. Details as to how many patients were children were not given. Initial testing showed that 40.6% of these patients had AMA, with reactivity against the major mitochondrial antigens (PDC-E2, BCOADC-E2, and OGDC-E2) [32]. By 24 months, only one subject remained positive for AMA directed against OGDC-E2 [32]. Similar data have been reported in the series of ALF patients from King’s College Hospital in London [24]. In that report, 13/47 (28%) sera from ALF patients tested positive for AMA, using the highly sensitive and specific MIT-3 (mitochondrial antigen 3) enzyme-linked immunosorbent assay (ELISA) [24]. Of interest, these 13 cases tested negative for AMA using IIF, indicating that the sensitivity for the detection of AMA largely depends on the methodology used [24].

Unlike the reported cases of transient AMA, liver histology in this case showed biliary damage. In our experience, ALF in paediatric patients is not associated with the presence of AMA. During a large-scale autoimmune-screening program over a 7-year period, only 2 out of 3808 Italian children with liver diseases of a variety of causes tested positive for AMA.

In conclusion, we report a case of AIH-2 with PBC-specific AMA positivity, documented both by IIF and mitochondrial antigen-specific immunoassays. Investigation of this peculiar case may give insight into the mechanisms responsible for the breakdown of immunological tolerance.

## Consent

Written informed consent was obtained from the patient’s parents for publication of this Case report and any accompanying images. A copy of the written consent is available for review by the Series Editor of this journal.

## Grant support:

DSS is supported by Liver Immunodiagnostics, KCL grant; AL is supported by AISF (Associazione Italiana per lo Studio del Fegato), Mario Coppo award; DPB is supported by a CSL award from the Higher Education Funding Council for England.

## Abbreviations

AIH, Autoimmune Hepatitis; ALF, Acute Liver Failure; AMA, Antimitochondrial Antibodies; anti-LKM-1, Anti-liver Kidney Microsome type 1 antibodies; AST, Aspartate amonitransferase; GGT, Gamma Glutamyl Transpeptidase activity; IIF, Indirect Immunofluorescence; OLT, Orthotopic liver transplant; PBC, Primary Biliary Cirrhosis; PDC-E2, E2 subunit of the pyruvate dehydrogenase complex.

## Competing interest

The author(s) declare that they have no competing interests'.

## Authors contributions

PI designed the study and had the overall supervision; PI, AL, DSS and DPB have written the first and subsequent drafts of the manuscript; PI, MGA, LF, GT conceived of the study, and participated in its design and coordination and helped to draft the manuscript. MGA, AL and DPB have carried out the immunoassays; MGA, MB, MC, RI, GT recruited biological material, collected data, and revised the manuscript. AS has performed histopathological assessment, immunohistochemical analysis and produced the photo of the liver tissue sections. All authors read and approved the final manuscript.
